# Postoperative Fractionated Stereotactic Radiosurgery to the Tumor Bed for Surgically Resected Brain Metastases

**DOI:** 10.7759/cureus.1279

**Published:** 2017-05-26

**Authors:** Ryan K Cleary, Jessica Meshman, Michael Dewan, Liping Du, Anthony J Cmelak, Guozhen Luo, Manuel Morales-Paliza, Kyle Weaver, Reid Thompson, Lola B Chambless, Albert Attia

**Affiliations:** 1 Department of Radiation Oncology, Vanderbilt University Medical Center; 2 Department of Neurological Surgery, Vanderbilt University Medical Center; 3 Center for Quantitative Sciences, Vanderbilt University School of Medicine

**Keywords:** stereotactic radiosurgery, brain metastases, fractionated radiotherapy, frameless stereotactic radiosurgery, fsrs

## Abstract

Introduction

Stereotactic radiosurgery (SRS) is increasingly used as an alternative to whole brain radiotherapy (WBRT) following surgical resection of brain metastases. We analyzed the outcomes of postoperative frameless fractionated stereotactic radiosurgery (fSRS) cases for surgically resected brain metastases at our institution.

Materials and Methods

We performed a retrospective review of 85 patients who underwent fSRS to 87 resection beds from 2006 - 2014 with a median follow-up of 6.4 months. Clinically relevant outcomes were assessed with analysis to determine predictors of these outcomes.

Results

The median target volume was 9.8 cm­^3^ (1.1 - 43.1 cm­^3^). The most frequently used fractionation scheme was 3,000 cGy in five fractions. The rates of local control (LC), distant brain failure (DBF), and overall survival (OS) at one-year were 87%, 52%, and 52%, respectively. Five patients (5.9%) experienced Grade >2 toxicity related to fSRS, including seizures (two), symptomatic radionecrosis (two), and potential treatment-related death (one). A multivariable analysis revealed that tumor volume (p < 0.001) and number of fractions (p < 0.001) were associated with LC, while recursive partitioning analysis (RPA) class (*p *< .0001), tumor volume (*p *= .0181), and the number of fractions (*p = *.0181) were associated with OS.

Conclusions

Postoperative fSRS for surgically resected brain metastases is well-tolerated and achieves durable LC. Further studies are needed to determine the optimal dose and fractionation for fSRS as well as to compare outcomes with WBRT.

## Introduction

Whole-brain radiation therapy (WBRT) became the standard of care following surgical resection of a single brain metastasis based on the results published by Patchell, et al. [1]. Their randomized trial showed decreased local and distant brain failure (DBF) rates after WBRT versus observation alone. Reduced incidence of neurological death was also seen with WBRT, though an overall survival (OS) benefit was not shown. Focal techniques, such as stereotactic radiosurgery (SRS), emerged as an alternative to WBRT in order to provide local control (LC) at the resection cavity, while sparing radiation to normal brain. Results from multiple series show LC on the order of 75-85% with DBF rates of approximately 40-60% at one year [2-3]. Fractionated stereotactic radiosurgery (fSRS) has been employed in treating surgical cavities of increasing size in an attempt to maintain LC while reducing the risk of treatment toxicity [4-7]. Herein, we report on the postoperative fSRS experience at Vanderbilt University Medical Center.

## Materials and methods

Between February 2006 and November 2014, 85 patients underwent surgical resection of a brain metastasis followed by fSRS. Two of these patients developed an additional brain metastasis at a separate site in the brain requiring surgical resection followed by adjuvant stereotactic radiation for a total of 87 treated resection cavities. Additional contemporaneous metastases discovered on imaging were treated with SRS according to the usual fashion.

Data regarding treatment of these patients were acquired retrospectively. Prior to the acquisition of data, Vanderbilt University Medical Center Institutional Review Board approval (#150276) was obtained. 

### Radiosurgery technique

Radiosurgery was performed with a Novalis Tx^TM^ linear accelerator-based radiosurgery platform (Varian Medical Systems, Palo Alto, CA). ExacTrac® (BrainLab, Munich, Germany) stereoscopic kV x-ray monitoring for patient localization was employed for all cases starting in 2009. Using this frameless method, patients were immobilized supine on the treatment table with a custom thermoplastic SRS mask. All patients underwent postoperative gadolinium-enhanced magnetic resonance imaging (MRI) of the brain within one to two days following surgery to assess the extent of the surgical resection. An additional 1-mm slice thickness contrasted MRI of the brain was performed within one to three weeks of radiosurgery for treatment planning purposes. Typically, fSRS was started within four weeks following the date of surgery. 

The edge of the resection cavity with any associated residual contrast enhancement was designated the tumor volume. Contouring was performed by the radiation oncologist and the operating neurosurgeon. Use of an expansion margin of 1-2 mm was performed at the discretion of the treating radiation oncologist. The prescription dose and fractionation were determined by the radiation oncologist with consideration for cavity size and surrounding normal tissue constraints.

### Follow-up

Patients received regular follow-up with the treating radiation oncologist every two to three months for the first year, then every three to six months thereafter. Neurological examination was performed at each visit. The Radiation Therapy Oncology Group (RTOG) grading system was used to assess toxicity. Treatment-related death was defined as death within 30 days of completion of radiosurgery. Determination of radionecrosis of the brain was made on MRI by the multidisciplinary neuro-oncology tumor board, which included the treating radiation oncologist, neurosurgeon, pathologist, and radiologist. Neurologic death was defined as intracranial progression at the time of death in the absence of systemic disease progression [1].

### Statistical analysis

Patient demographics and disease and treatment characteristics were summarized using frequencies and relative frequencies for categorical variables. Medians and ranges were used for continuous variables. Times to death, local recurrence (LR), and DBF were calculated from the date of radiosurgery to the date of the defined event. For OS, the event was death and those who were alive at the last follow-up were censored. For LC (defined as the absence of radiographic evidence of disease recurrence within 1 cm of the surgical resection cavity), the event of interest was LR, while death without LR was considered as a competing risk; those with neither LR nor death were censored at the last follow-up. For distant control (defined as lack of development of new brain metastases or leptomeningeal disease outside of the treatment volume), the event of interest was DBF, and death without DBF was as a competing risk; those with neither DBF nor death were censored at the last follow-up. For OS, the data for the 85 patients who were considered independent were used. The Kaplan-Meier method was used to estimate the survival rates, and multivariable Cox regression with robust standard errors was performed to estimate the adjusted hazard ratios (HR). For LC and distant control, competing risk survival analysis using Fine and Gray’s method [8-9] was performed using the data for the 87 treated resection cavities that were considered independent. All the multivariable regression models were pre-specified to increase the precision of estimation and avoid potential confounding. Covariates, such as age, recursive partitioning analysis (RPA) class, primary tumor site, tumor volume, and the number of fractions (2, 3, and 4-5) were included in the Cox regression model for OS. Age, sex, RPA class, primary tumor site, tumor volume, and the number of fractions (2, 3, and 4-5) were included in the local control multivariable model. In addition to these covariates, margin use or not, the number of tumors in the brain and systemic disease status were included in the model for distant control. The two-sided nominal level of 0.05 was considered as statistically significant for all the tests. All statistical analyses were performed using software R version 3. 2.4, including packages “ survival”, “Hmisc”, “rms”, and “mstate”.

## Results

At our institution, 85 patients underwent postoperative fSRS following surgical resection of a brain metastasis between February 2006 and November 2014. Two patients underwent resection of an additional brain metastasis at a different time point for a total of 87 resection cavities. Baseline patient characteristics are listed in Table 1. The median age of our patients was 58.9 years (range: 30.9 - 85.3). Over half of our patients were RPA class 2 (52.9%). Non-small cell lung cancer (NSCLC) (40.0%) and melanoma (21.2%) were the most common primary histologic subtypes treated. Sixty-two patients (72.9%) had a single resected brain metastasis, whereas the remaining patients had additional brain metastases managed with SRS. Four patients (4.7%) had received prior WBRT.

**Table 1:** Patient Characteristics RPA: recursive partitioning analysis; NSCLC: non-small cell lung cancer; SCLC: small cell lung cancer; WBRT: whole brain radiotherapy. Values are number (percentage) unless otherwise noted.

Variable	Patients
No. of patients	85
Age (median in y)	58.9
Sex	
Male	52 (61.2%)
Female	33 (38.8%)
RPA Class	
1	24 (28.2%)
2	45 (52.9%)
3	16 (18.8%)
Primary	
Breast	8 (9.4%)
Colorectal	3 (3.5%)
Head and neck	1 (1.2%)
Melanoma	18 (21.2%)
NSCLC	34 (40.0%)
Renal cell	9 (10.6%)
SCLC	2 (2.3%)
Other	10 (11.8%)
Number of brain metastases	
1	62 (72.9%)
2	9 (10.6%)
3	6 (7.1%)
4+	8 (9.4%)
Systemic disease burden	
None	36 (42.3%)
Oligometastatic (< 5 sites)	18 (21.2%)
Widespread	31 (36.4%)
Systemic disease status	
Stable	43 (50.6%)
Progressive	40 (47.1%)
Unknown	2 (2.4%)
Prior WBRT	
Yes	4 (4.7%)
No	81 (95.3%)

All resection cavities received radiosurgery delivered in two to five fractions (See Table 2 for treatment characteristics). The median size of the resection cavities in our series was 9.8 cc (range: 1.1 - 43.1 cc). The median prescription dose was 3,000 cGy (range: 1,800 - 3,500). 

**Table 2:** Treatment Parameters cGy - centigray Values are median (range) or number (percentage). *There is one missing datum, making 86 known tumor volumes.

Parameter	Value
Tumor volume (cm^3^)	9.8 (1.1 - 43.1)*
Fractions	
2	17 (19.5%)
3	27 (31.0%)
4	5 (5.6%)
5	38 (43.7%)
Prescribed dose (cGy)	3,000 (1,800 - 3,500)
Maximum dose (cGy)	3,549 (2,134 - 4,388)
Margin use	
Yes	36 (41.3%)
No	51 (58.7%)

**Figure 1:**
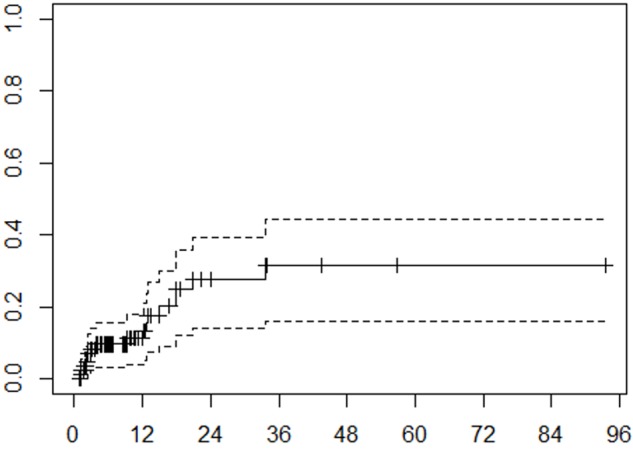
Local recurrence Cumulative incidence function of local recurrence. Months from treatment on x-axis, cumulative incidence on y-axis. Tick marks on the solid curve represent censored data. Dashed lines represent 95% confidence interval.

The median follow-up time for our cohort was 6.4 months (range: 0.6 - 93.5). The estimated six-month and one-year cumulative incidence rates for LR in the presence of competing risk were 10% and 13%, respectively (Figure 1). Statistically significant predictors of LC from the multivariable competing risk survival analysis were tumor volume (p = < .0001) and number of fractions (p = < .0001). The analysis also revealed a statistically significant effect modification of tumor volumes on the number of fractions; the effect of increasing number of fractions on improving local control was most important with larger tumor volumes.  

**Figure 2:**
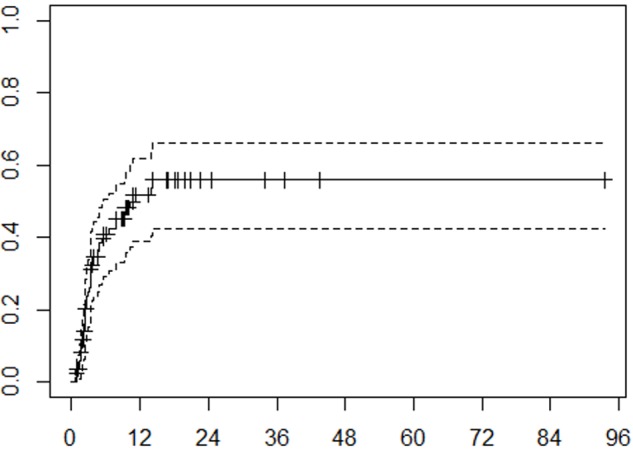
Distant brain failure Cumulative incidence function of distant brain failure. Months from treatment on x-axis, cumulative incidence on y-axis. Tick marks on the solid curve represent censored data. Dashed lines represent 95% confidence interval.

The estimated cumulative incidence rates for DBF at six months and one year were 41% and 52%, respectively (Figure 2). Ten patients experienced leptomeningeal failure while the remainder had new brain metastases. Multivariable analysis revealed that tumor volume (p = .0222) and systemic disease status (p = .0161) were statistically significantly associated with DBF.    

**Figure 3:**
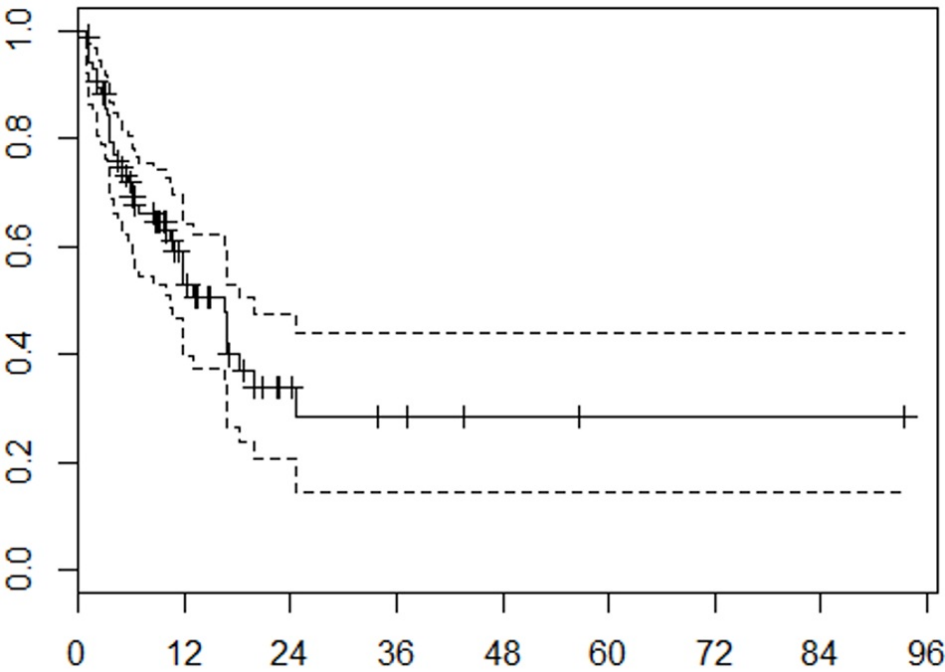
Overall survival Kaplan-Meier analysis of overall survival. Months from treatment on x-axis, probability on y-axis. Tick marks on the solid curve represent censored data. Dashed lines represent 95% confidence interval.

The median OS for our patients was 13.0 months. The estimated actuarial OS rates at six months and one year were 68% and 52%, respectively (Figure 3). RPA class (p < .0001), tumor volume (p = .0181), and number of fractions (p = .0181) were statistically significantly associated with OS on multivariable analysis. 

Five patients (5.9%) experienced Grade > 2 toxicity related to fSRS. One patient died within 30 days of receiving fSRS of 3,000 cGy in five fractions to a 5.6 cc cerebellar resection cavity. His course was complicated by vasogenic edema and disease progression causing obstructive hydrocephalus which eventually led to his demise. Another two patients experienced seizures shortly after undergoing radiosurgery (2.4%). Symptomatic radionecrosis was seen in two patients over the course of follow-up (2.4%). Neurologic death was seen in 16 patients (18.8%).

## Discussion

Whole brain radiotherapy has been shown to reduce the rates of LR and DBF following surgical resection of a single brain metastasis [1]. Patients in the Patchell trial who underwent WBRT were less likely to experience LR (10% vs. 46%, respectively), DBF (14% vs. 37%), or recurrence anywhere in the brain (18% vs. 70%) versus observation. However, no OS benefit was seen with the addition of WBRT. Moreover, WBRT has been associated with neurocognitive impairment [10]. Therefore, patients are increasingly being managed in the postoperative setting with radiosurgery alone to the surgical resection cavity and any remaining sites of intracranial metastatic disease. Jensen, et al. showed that high-resolution MRI and cavity-directed radiosurgery could be used following resection of a brain metastasis to spare over 50% of patients from undergoing WBRT at one year [11].    

Traditionally, patients have been treated with single fraction SRS with recent series showing local control at one year from 73-94% [3, 12-14]. A prospective, randomized trial of single-fraction SRS to the surgical cavity versus observation alone after brain metastasis resection was performed at the MD Anderson Cancer Center (MDACC) and recently presented at the American Society of Radiation Oncology (ASTRO) 2016 Annual Meeting [15]. This trial showed improved one-year LC for postoperative SRS as compared to observation alone (72% vs. 45%, respectively), although survival was the same for both groups. Many centers are moving toward fSRS for larger resection cavities, given the concern for normal tissue toxicity. Our retrospective study of patients treated with fSRS following surgical resection of a brain metastasis is one of the larger series in the literature and shows excellent local control (87%) at one year. This is in line with several other published series of fSRS which report one-year local control of 79-93%. [2, 4, 6-7]. Our median cavity size was similar to these series at 9.8 cc, and cavities up to 43 cc in size were treated safely. Distant brain failure was 48% at one year, which is also comparable to these series. Although one patient died shortly after fSRS as a possible complication of treatment versus disease progression, the treatment was generally well-tolerated with few side effects, including only two cases of symptomatic radionecrosis. 

The most intriguing finding from our study was the association between the increasing number of fractions and improved local tumor control. This effect appears to be most pronounced with larger tumor volumes. One explanation for this finding is that through increasing the number of fractions, a larger biologically effective dose (BED) was delivered to the cavity as compared to administering a smaller number of fractions due to physician concern for long-term toxicity with larger fraction sizes and/or fewer fractions, especially as cavity size increases. Previous single fraction SRS series for intact brain metastases have shown that local control decreases with increasing tumor volume [16-17]. This has been noted in series of brain metastases treated with multimodality therapy, including fSRS [7]. Notably, the incidence of radionecrosis after single-fraction SRS has been shown to increase significantly when the volume receiving 12 Gy is > 8 cm^2^ [18]. Fractionated stereotactic radiosurgery offers the ability to increase the dose to the cavity while minimizing side effects as the rate of symptomatic radionecrosis was only 2.4% in the current series. The most frequently used fractionation scheme for our patients was 600 cGy x five fractions delivered on consecutive days. The BED_10_ for this scheme is roughly equivalent to 17.5 Gy delivered in a single fraction. As a point of reference, the recently completed Phase III Randomized Study of Post-Surgical Stereotactic Radiosurgery Versus Whole-Brain Radiotherapy in Patients with Resected Metastatic Brain Metastases (NCCTG-N107C) trial comparing post-surgical SRS with WBRT for resected metastatic brain disease dictated single-fraction prescription doses from 12-20 Gy depending on cavity size [19]. Although the initial results from this trial presented at ASTRO 2016 showed similar OS and improved cognitive outcomes with postoperative SRS versus WBRT, the surgical bed control rate in the SRS arm was significantly worse at 12 months as compared to WBRT (55.6% vs 78.2%, respectively). The numerically higher incidence of surgical bed failure in the single-fraction SRS arm of NCCTG-N107C and in the prospective MDACC trial, as compared to the current series, suggests that further studies are needed to determine if fSRS may provide the means to maintain local control while minimizing side effects, especially with larger cavity sizes. 

The limitations of our study include the short median follow-up time, as well as the number of patients examined. Both of these limit the power of our study to determine predictors of patient outcome via multivariable analysis. Additionally, our study is limited by its retrospective nature without a control group treated with alternate modalities. However, our study provides further evidence regarding the efficacy and safety of postoperative fSRS for the treatment of brain metastases.

## Conclusions

Stereotactic radiosurgery is an emerging alternative to WBRT following surgical resection of brain metastases. We performed a retrospective review of patients treated at our institution with frameless fSRS after undergoing neurosurgical extirpation of an intracranial metastasis. Results from our study show that fSRS to the postoperative resection cavity with a median dose of 30 Gy in five fractions provides excellent LC (87% at one year) with minimal neurotoxicity, thus sparing many patients the untoward neurocognitive effects of WBRT. Additional multivariate analysis revealed that tumor volume and number of fractions are associated with LC, suggesting that fractionation may play a role in improving LC rates in these patients. Further studies are needed determine the optimal dose and fractionation for multimodality treatment of brain metastases with fSRS.
